# Olfactory Sensitivity Is Associated with Body Mass Index and Polymorphism in the Voltage-Gated Potassium Channels *Kv1.3*

**DOI:** 10.3390/nu14234986

**Published:** 2022-11-24

**Authors:** Melania Melis, Iole Tomassini Barbarossa, Roberto Crnjar, Giorgia Sollai

**Affiliations:** Department of Biomedical Sciences, University of Cagliari, 09042 Monserrato, CA, Italy

**Keywords:** smell, olfactory dysfunction, BMI, voltage-gated potassium channels Kv1.3, sex, Sniffin’ Sticks

## Abstract

Smell strongly contributes to food choice and its hedonistic evaluation. A reduction or loss of smell has been related to malnutrition problems, resulting in excessive weight loss or gain. Voltage-gated potassium channels Kv1.3 are widely expressed in the olfactory bulb, and contribute mainly to the value of the resting membrane potential and to the frequency of action potentials. Mutations in the *Kv1.3* gene are associated with alterations in glycemic homeostasis and olfactory sensitivity. We evaluated the olfactory performance in 102 healthy subjects and its association with BMI and polymorphism in the human *Kv1.3* gene. Olfactory performance, based on the olfactory threshold, discrimination and identification scores and their summed score (TDI), was measured using the “Sniffin’ Sticks” test. Subjects were genotyped for the *rs2821557* polymorphism of the *Kv1.3* gene, whose major allele T was associated with a super-smeller phenotype, lower plasma glucose levels and resistance to diet-induced obesity as compared with the minor allele C. Based on the *Kv1.3* genotype, the TDI and I olfactory scores obtained by the subjects were the following: TT > TC > CC. Subjects who were TT homozygous or heterozygous exhibited lower BMIs and reached higher olfactory scores than those with the CC genotype. The results were sex-dependent: heterozygous females performed better than heterozygous males. These findings show an inverse relationship between olfactory function and BMI, and a significant effect of the *Kv1.3* genotypes on the olfactory functions and on the BMIs of the subjects. Finally, they suggest that the sex-related differences in the olfactory function can be partially ascribed to the *Kv1.3* gene’s polymorphism.

## 1. Introduction

In humans, the olfactory function strongly influences the quality of life, playing an important role in eating behavior, in the ability to detect odors that signal the presence of dangers (e.g.,: gas, smoke, spoiled food), in social communication (reproductive behavior, mother-infant recognition, identification of potential mating partners) and in personal hygiene [1,2,3]. Olfaction contributes to nutritional health and food enjoyment by mediating the perception of food odors. Most people who exhibit olfactory impairment report that food is less flavorful and less enjoyable, and these conditions consequently change their eating habits. In general, these subjects, who present a tendency to obesity, report an increased intake of more palatable foods, such as sweet and high-fat foods over fruits and vegetables, as well as a larger use of condiments and spices [4,5,6], to compensate for the reduced gratification that comes from receiving less olfactory stimulation.

Individuals can be classified as normosmic, hyposmic or anosmic depending on whether they show a normal, reduced or absent ability to detect odors; anosmia can be general or specific [2,7]. The causes of this individual variability are many and can be traced back to personal experience, environmental factors and genetic factors [8,9,10,11,12,13,14,15,16,17,18]. This aspect becomes even more complex if we consider that human perceptions of odors differ enormously among individuals, especially in terms of intensity and perceived pleasantness [12,19,20,21,22,23,24,25].

The axons of olfactory sensory neurons (OSNs) project to the olfactory bulb, where they make synaptic contact with mitral-cell dendrites in specific regions called glomeruli. The voltage-gated potassium channel Kv1.3 has been shown to be highly expressed in the olfactory bulb (OB), where it carries a large proportion of the outward current in mitral and granule cells. In general, voltage-gated potassium channels in the OB are thought to contribute to the resting membrane potential, determine the frequency of repetitive firing and influence the interspike interval. In addition to being linked to energy metabolism by acting on plasma glucose levels and insulin sensitivity, the function of the channel Kv1.3 has been shown to be associated with the olfactory performances of individuals [26,27,28]. Furthermore, it was found that of the five single-nucleotide polymorphisms of the gene encoding for potassium channels Kv1.3, only T-1645C has a role in modulating the activity of the channel and its effects [27].

The functionally relevant *rs2821557* (*T/C*) polymorphism of the *Kv1.3* gene has been associated with alterations in glycemic homeostasis and olfactory sensitivity. In particular, the major allele T was associated with a super-smeller phenotype, lower plasma glucose levels and resistance to diet-induced obesity as compared with the minor allele C [26,29].

Several studies have reported a relationship between olfactory function, body weight and metabolic status [30]. On the one hand, overweight or obese people have a reduced olfactory function, as shown by the fact that they obtain lower scores for odor threshold, discrimination and identification [31,32,33]; on the other hand, subjects with a reduced sense of smell show higher BMIs [33,34]. Although it is commonly accepted that females perform better than males in their olfactory abilities, some studies on a large number of individuals report that there are no sex-related differences in the olfactory function of individuals [35,36,37], leaving the topic as a matter of debate. The elements considered to be potentially responsible for generating sex-related differences in olfactory abilities include: neuroendocrine factors (e.g., fluctuations associated with the menstrual cycle or estrogen levels) [38,39,40,41], social factors (females appear to be more interested in olfactory stimuli and are more familiar with odors) [42,43,44] and cognitive factors (women perform better than men in episodic olfactory memory) [43,45].

Based on these considerations, in this study we evaluated: first, the association between the *rs2824557* polymorphism encoding for the voltage-gated potassium channel (Kv1.3) and the overall olfactory performance, odor threshold, odor discrimination and odor identification; second, the effect of the Kv1.3 genotypes on the BMIs of the subjects. Furthermore, by considering that genetic aspects have not yet been evaluated among the factors responsible for sex-related differences, we assessed the presence of any different effects of this polymorphism on olfactory function and BMI in females and males separately. Finally, we looked for a correlation between BMI and olfactory scores, as the sense of smell influences food choices and intake, which in turn influence body weight and BMI.

## 2. Materials and Methods

### 2.1. Subjects

One hundred and two Caucasian volunteers (59 F, 43 M; age 45,82 ± 1.90 years) were recruited in the metropolitan area of Cagliari (Sardinia, Italy) by means of a public announcement at the local University. For each subject, exclusion criteria were the presence of neurological or psychiatric diseases, pregnancy or lactation, history of cancer, head trauma, sinusitis or nasal sept disorders. Subjects who claimed to have had allergic responses or nasal congestion prior to the scent tests were excluded from the study. All participants were fragrance-free and had to fast for at least two hours before the test.

The ratio between the subject’s weight and the square of their height (kg/m^2^) was used to calculate the BMI and to classify each subject for his/her weight status.

The study was carried out in conformity with the Declaration of Helsinki, and the local Ethics Committee authorized it. Each individual was instructed on the scope of the study and the experimental procedures before being tested, and was asked to sign an informed consent form.

### 2.2. Olfactory Sensitivity Screening

We used the standardized “Sniffin’ Sticks” test battery (Burghart Instruments, Wedel, Germany) with three subtests for olfactory threshold (T-test), olfactory discrimination (D-test) and olfactory identification (I-test) to assess the orthonasal olfactory functions of individuals [46].

The investigator had 48 pens available for determining the olfactory threshold, divided into 16 triplets: two pens contained a solvent and the third was soaked with n-butanol at increasing concentrations. The triplets were presented in increasing order until the subject selected twice in a row the pen containing n-butanol in the same triplet. This was the starting point, and it denoted the first reversal, in which the triplets were displayed in decreasing order of n-butanol dilution. The dilution sequence in which the triplets were presented was reversed whenever the subject did not recognize the target pen. When the seventh reversal occurred, the experiment stopped, and the threshold score was determined by the average of the previous four reversals. Furthermore, the experimenter had 16 triplets available for determining odor discrimination, each consisting of two pens filled with the same odor and one filled with a different one (target pen). The objective was to find the target pen. From 0 to 16, the obtained score corresponded to the number of right answers. Finally, 16 pens were used to determine the identification of odors, containing as many aromas as possible familiar to the subjects. Each pen had four different options for the subject to choose from. The score (from 0 to 16) corresponded to the number of correct identifications.

The total TDI was calculated by adding the scores obtained with the T-test, D-test and I-test, and was used to classify persons as normosmic or hyposmic based on their overall olfactory performance. Subjects could also be categorized by olfactory threshold, discrimination and identification based on the results of the T-test, D-test and I-test [47].

### 2.3. Genetic Analyses

The “QIAamp^®^ DNA” Mini Kit (Qiagen srl, Milan, Italy) was used to extract DNA from saliva samples, in accordance with the procedure reported in the manufacturer’s instructions. Subjects were genotyped for the *rs2821557* (*T/C*) polymorphism of the human potassium channel *Kv1.3* gene using the TaqMan^®^ SNP Genotyping Assay technique by means of the assay with code: C_16121408_10 Assay, specific for the SNP of interest (Applied Biosystems by Life-Technologies, Monza, Italy).

After PCRs, the fluorescences of the plates were read by the sequence-detector system at 60 °C for 1 min, and the results were analyzed by allelic discrimination of the sequence-detector software (Applied Biosystems). The reactions included three positive controls (one for each genotype), two negative controls and two replicates.

### 2.4. Data Analyses

One-way ANOVA was used to analyze: (a) the effects of the *Kv1.3* genotype on the TDI olfactory scores obtained by the subjects; (b) the effects of the *Kv1.3* genotype on the BMIs of the subjects.

One-way multivariate analysis of variance (MANOVA) was used to analyze the differences of the T, D and I scores according to *Kv1.3*.

Two-way ANOVA was used to verify for a significant interaction between sex × *Kv1.3* genotype on the scores obtained with the T-test, D-test and I-test and their TDI sum and on the BMIs of the subjects.

Three-way ANOVA was used to verify for a significant interaction among sex × *Kv1.3* genotype × TDI olfactory status on the BMIs of the subjects.

The assumptions of homogeneity of variance and normality were tested on the data. Post-hoc comparisons were made using Fisher’s least-significant-difference (LSD) test; if the assumption of homogeneity of variance was violated, we applied Duncan’s test. STATISTICA for WINDOWS was used to conduct statistical analysis (version 7.0; StatSoft Inc., Tulsa, OK, USA). *p* values < 0.05 were considered significant.

Fisher’s method (Genepop software version 4.2; http://genepop.curtin.edu.au/genepop_op3.html) [48] was used to analyze differences on genotype distributions and allele frequencies at the *Kv1.3* locus: (a) between subjects classified as normosmic or hyposmic for the TDI olfactory status, and individually for the T, D and I status; (b) between subjects classified as normal weight or overweight according to their BMI status.

The Pearson’s correlation coefficient was used to evaluate the association between BMI and olfactory scores, considering females and males both together and separately. Statistical analyses were performed using GraphPad Prism 6 (GraphPad Software, San Diego, CA, USA). *p* values < 0.05 were considered significant.

## 3. Results

### 3.1. Olfactory Scores and Kv1.3 Genotype

**Figure 1:**
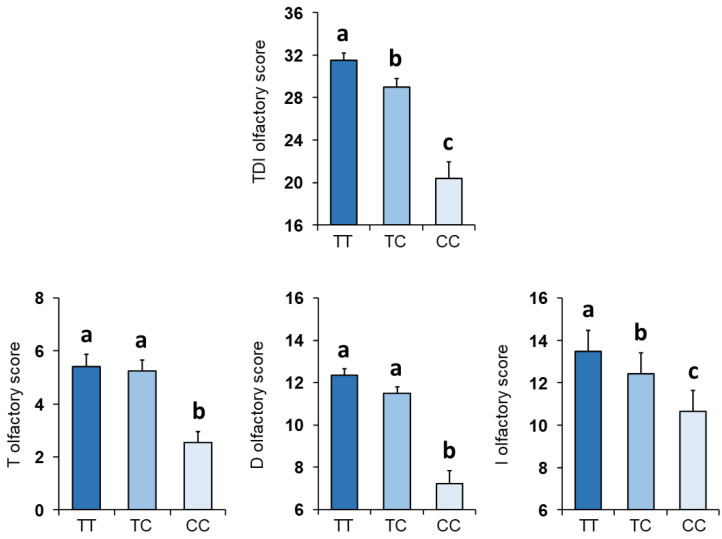
Mean (± SEM) values of the threshold (T), discrimination (D) and identification (I) olfactory scores and their summed TDI olfactory scores obtained by subjects according to genotypes of the *Kv1.3* locus (*n* = 102; 45 TT, 40 TC, 17 CC). Different letters indicate a significant difference (*p* < 0.05; Fisher’s LSD test).

Molecular analyses for the *rs2821557* (*T/C*) polymorphism of the *Kv1.3* gene allowed for us to identify the genotype of 102 subjects: 45 were TT homozygous, 40 were heterozygous and 17 were CC homozygous. Figure 1 shows a significant relationship between the *rs2821557* (*T/C*) polymorphism of the *Kv1.3* locus and the TDI olfactory score (*F* _2,99_ = 27.52; *p* < 0.0001). One-way MANOVA also revealed a significant effect of the *Kv1.3* genotype on the T, D and I olfactory scores (*F* _6,194_ = 11.71; *p* < 0.0001) (Figure 1). Post-hoc comparisons showed that the subjects that were homozygous for the T allele or were heterozygous exhibited T and D olfactory scores higher than those who were homozygous for the C allele (*p* < 0.001; Fisher’s LSD test); instead, for the I score, subjects with the TT genotype scored higher than those with both the CC (*p* < 0.0001; Fisher’s LSD test) and TC genotypes (*p* = 0.038; Fisher’s LSD test), and subjects who were homozygous for the C allele also scored lower than heterozygous ones (*p* = 0.008; Fisher’s LSD test).

Genotype distributions and allele frequencies for the *rs2821557* (*T/C*) polymorphism of the *Kv1.3* gene according to TDI, T, D and I olfactory statuses are shown in Table 1. Subjects classified as normosmic and hyposmic based on their TDI olfactory scores differed on the basis of genotype distribution (*χ2* = 21.64, *p* < 0.0001; Fisher’s method) and allelic frequencies (*χ2* = 17.90, *p* = 0.0001; Fisher’s method). The results indicate that significant differences, based on the genotype distribution and allele frequencies of the *Kv1.3* locus, also existed when subjects were classified as normosmic or hyposmic by means of the scored obtained with T, D and I subtests (Genotype: T *χ2* = 9.46, *p* = 0.0088; D *χ2* = 31.89, *p* < 0.0001; I *χ2* = 13.78, *p* = 0.001. Allele frequency: T *χ2* = 10.40, *p* = 0.0055; D *χ2* = 31.88, *p* < 0.0001; I *χ2* = 15.75, *p* = 0.0004; Fisher’s method).

**Figure 2:**
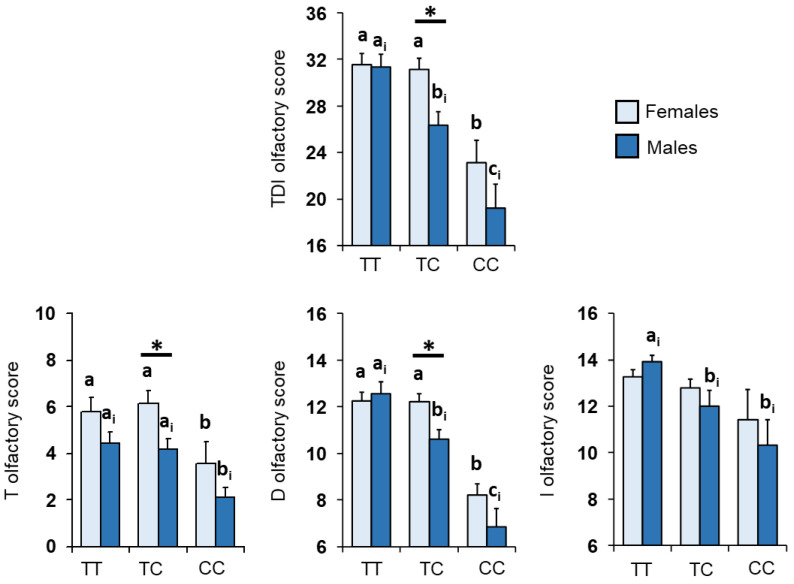
Mean (± SEM) values of the threshold (T), discrimination (D) and identification (I) olfactory scores and their summed TDI olfactory scores obtained by females (*n* = 59: 32 TT, 22 TC, 5 CC) and males (*n* = 43: 13 TT, 18 TC, 12 CC) according to the genotypes of the *Kv1.3* locus. Different letters indicate a significant difference: a,b for females (TDI: *p* < 0.005; T: *p* < 0.05; D: *p* < 0.0005; Fisher’s LSD test), a_i_–c_i_ for males (TDI and D: *p* < 0.005; T and I: *p* < 0.05; Fisher’s LSD test). (*) indicates significant differences between females and males with the same genotype (*p* < 0.05; Fisher’s LSD test).

The mean values ± SEM of the T, D and I olfactory scores and their summed TDI scores obtained by female and male subjects are shown in Figure 2. In females, post-hoc comparisons subsequent to two-way ANOVA revealed that subjects who were homozygous for the T allele or were heterozygous scored on the TDI, T and D tests higher than those who were homozygous for the C allele (TDI: *p* < 0.005; T: *p* < 0.05; D: *p* < 0.0005; Fisher’s LSD test), while no differences in the I olfactory scores were found in relation to genotype (*p* > 0.05; Fisher’s LSD test). Male subjects with the CC genotype scored lower than those with TT homozygotes in all olfactory scores considered (TDI, D and I: *p* < 0.0005; T: *p* = 0.026; Fisher’s LSD test) and compared with the heterozygotes-only in the TDI and D scores (*p* < 0.0005; Fisher’s LSD test); TT homozygotes also obtained TDI, D and I scores higher than heterozygotes (TDI and D: *p* < 0.005; I: *p* = 0.023; Fisher’s LSD test). Finally, a sex-related difference was found among heterozygous subjects, with females obtaining TDI, T and D scores higher than males (*p* < 0.02; Fisher’s LSD test).

### 3.2. BMI and Kv1.3 Genotype

**Figure 3:**
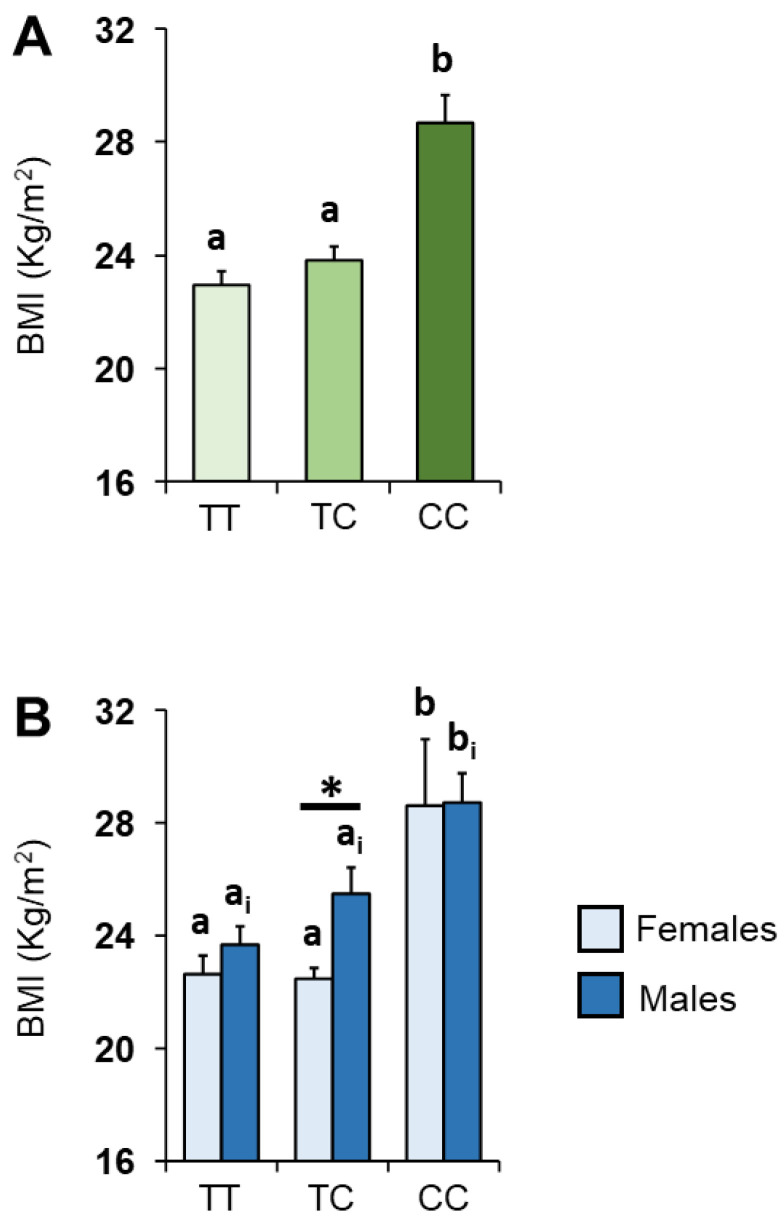
Mean (± SEM) values of the BMIs determined in healthy subjects considered all together (**A**) and separately for females and males (**B**) according to genotypes of the Kv1.3 locus. (**A**) Different letters indicate a significant difference (*p* < 0.0001; Fisher’s LSD test). (**B**) Different letters indicate a significant difference: a,b for females (*p* < 0.0005; Fisher’s LSD test), a_i_,b_i_ for males (*p* < 0.05; Fisher’s LSD test). Asterisk indicates a significant difference between females and males with the same genotype (*p* = 0.005; Fisher’s LSD test). (*) indicates significant differences between females and males with the same genotype (*p* < 0.01; Fisher’s LSD test).

The mean values ± SEM of the BMIs determined in the subjects according to the *rs2821557* polymorphism of the *Kv1.3* gene are shown in Figure 3A. One-way ANOVA revealed that BMIs of subjects who were homozygous for the C allele were significantly higher than those of the heterozygous or homozygous groups for the T allele (*F* _2,96_ = 18.01; *p* < 0.0001). Figure 3B shows the same data according to sex. Post-hoc comparisons subsequent to two-way ANOVA revealed that individuals with TT or TC genotype showed BMIs lower than those with the CC genotype (females *p* < 0.0005; males *p* ≤ 0.01; Fisher’s LSD test). In addition, pairwise comparison revealed that the BMIs of females with the TC genotype were significantly lower than those of males with the same genotype (*p* < 0.005; Fisher’s test LSD).

Genotype distributions and allele frequencies for the *rs2821557* (*T/C*) polymorphism of the *Kv1.3* gene according to BMI status are shown in Table 2. The results indicate that significant differences, based on the genotype distribution and allele frequencies of the *Kv1.3* locus, also existed when the subjects were classified as normal weight (NW) or overweight (OW) by means of their BMI (Genotype: NW *χ2* = 9.46, *p* = 0.0088; OW *χ2* = 31.89, *p* < 0.0001. Allele frequency: NW *χ2* = 10.40, *p* = 0.0055; OW *χ2* = 31.88, *p* < 0.0001; Fisher’s method).

**Figure 4:**
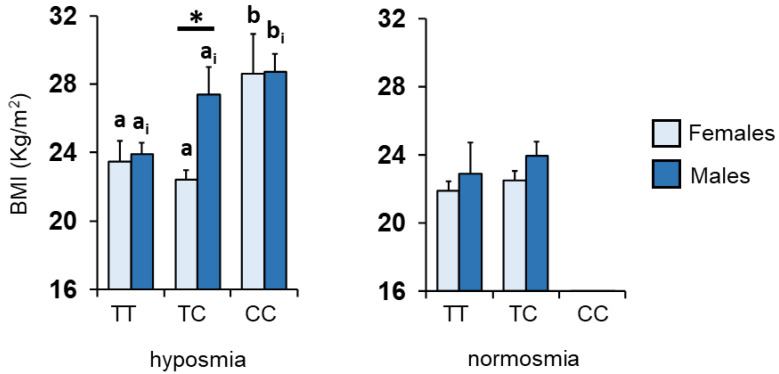
Mean (±SE) values of the BMIs determined in females and males according to their TDI olfactory statuses and to genotypes of the Kv1.3 locus. Different letters indicate a significant difference: a,b for females (*p* < 0.005; Fisher’s LSD test), a_i_,b_i_ for males (*p* < 0.05; Fisher’s LSD test). Asterisk indicates a significant difference between females and males with the same genotype (*p* = 0.0015; Fisher’s LSD test). (*) indicates significant differences between females and males with the same genotype (*p* < 0.01; Fisher’s LSD test).

The mean values ± SEM of the BMIs determined in the subjects according to the *rs2821557* polymorphism of the *Kv1.3* gene, sex and TDI olfactory status are shown in Figure 4. A pairwise comparison subsequent to three-way ANOVA showed, in hyposmic subjects, that females homozygous for the C allele exhibited a higher BMI than subjects that were TT homozygous or heterozygous (*p* < 0.005; Fisher’s LSD test), and that males who were heterozygous or CC homozygous exhibited a higher BMI than subjects that were homozygous for the T allele (*p* < 0.05; Fisher’s LSD test). Instead, no difference was observed between genotype and sex among normosmic individuals (*p* > 0.05; Fisher’s LSD test).

**Figure 5:**
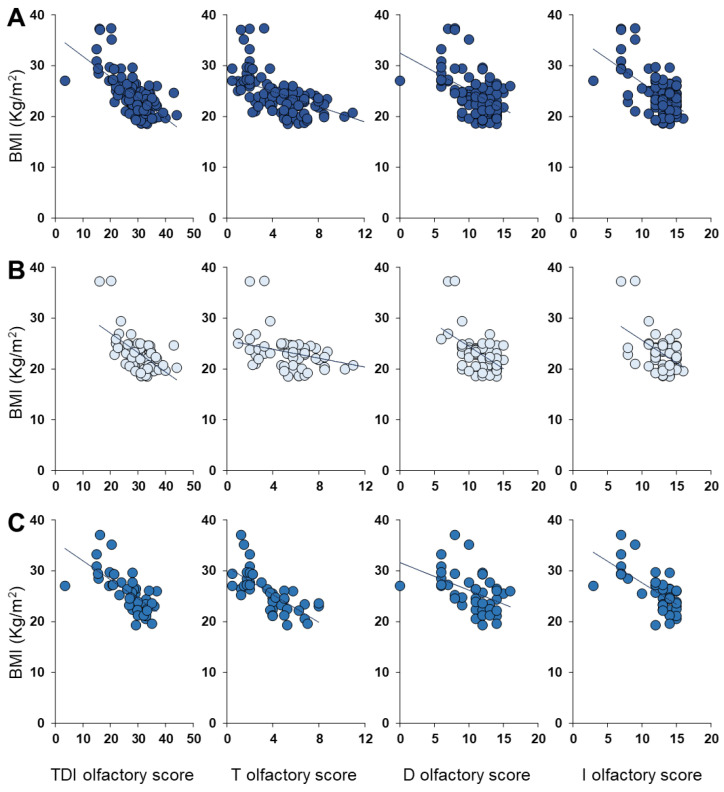
Correlation analysis between BMIs and TDI, T, D and I olfactory scores obtained by individuals of both sexes together (**A**), only females (**B**) or only males (**C**).

Pearson’s correlation test was used to check for a correlation between BMIs and olfactory scores obtained when considering subjects all together or females and males separately. The results shown in Figure 5 indicate that a significant negative correlation exists between the BMI of each individual and his/her TDI olfactory score (anyone Pearson’s *r* = −0.67, *p* < 0.0001; females Pearson’s *r* = −0.58, *p* < 0.0001; males Pearson’s *r* = −0.69, *p* < 0.0001). Negative correlations were also found between BMI and T olfactory score (anyone Pearson’s *r* = −0.54, *p* < 0.0001; females Pearson’s *r* = −0.37, *p* = 0.0042; males Pearson’s *r* = −0.71, *p* < 0.0001), D olfactory score (both sexes Pearson’s *r* = −0.51, *p* < 0.0001; females Pearson’s *r* = −0.53, *p* < 0.0001; males Pearson’s *r* = −0.45, *p* = 0.0023) and I olfactory score (both sexes Pearson’s *r* = −0.58, *p* < 0.0001; females Pearson’s *r* = −0.48, *p* = 0.0001; males Pearson’s *r* = −0.66, *p* < 0.0001).

## 4. Discussion

Information from the olfactory system plays an important role in food choices, and consequently in food intake. Recent studies have also shown that the olfactory system is able to perceive odors not only from the external environment, but also from the internal one, thus having a relevant function as a metabolic sensor [49]. In particular, the olfactory bulb seems to assist the function of the hypothalamus in controlling eating behavior. In fact, the hypothalamus induces feeding when nutrients are scarce or the levels of hormones such as ghrelin are high; on the contrary, it suppresses feeding in response to the abundance of nutrients, insulin and leptin [49,50,51]. Interestingly, olfactory structures such as the olfactory epithelium, olfactory bulb and piriformis cortex express high levels of hypothalamic-like hormone receptors (for ghrelin, leptin, insulin, CCK), causing the olfactory system to be considered an active sensor of signals from the internal environment [30].

Among the metabolic sensors, the voltage-dependent potassium channels Kv1.3 are of particular importance as, in addition to being expressed in muscle and adipose tissue, they are particularly abundant on the plasma membrane of the mitral and granular cells of the olfactory bulb. Here they perform a variety of functions, such as: contributing to the resting membrane potential, determining the repetitive frequency of action potentials, influencing the interspike interval, altering longevity and reducing adiposity by acting on metabolism [26,27,28,29,52,53,54,55,56]. In addition, they regulate energy homeostasis and body weight, peripheral insulin sensitivity, the migration of GLUT4 on the plasma membrane and the uptake of glucose and the olfactory threshold and odor discrimination. [26,27,28,29,52,53,54,55,56].

Based on these considerations, the main objective of this study was to evaluate whether there is a relationship between olfactory performance and *rs2821557* (*T*/*C*) polymorphism of the human gene that codes for potassium channels *Kv1.3*. The results we obtained show a significant relationship between Kv1.3 polymorphism and the T, D and I olfactory scores and their summed TDI score. In particular, the subjects who achieved the highest olfactory scores were homozygous for the wild T allele or heterozygous, while those who were homozygous for the C allele obtained significantly lower scores. These data confirm a previous study showing that individuals with the CC genotype achieved lower TDI scores than those who were TT homozygous or heterozygous [26]. They are also in agreement with an earlier study on mice: the experimental removal of the gene for Kv1.3 produced animals that were “super-smellers”, presenting a lower threshold of olfactory perception and an increased ability to discriminate between similar smells [52]. On the other hand, they highlight that the phenotypic differences related to the genotype of the *Kv1.3* channels are not limited to the TDI olfactory score, as they also affect the ability to identify odors. In our panel we observed that, between subjects classified as normosmic or hyposmic, there are different genotype distributions and allele frequencies: a higher orthonasal olfactory performance is associated with the genotype TT and allele T, while the CC genotype and C allele are associated with a hyposmic condition. It seems that homozygosity for the C allele leads to a gain in the functionality of Kv1.3 [27]. This means that the outward potassium current increases, resulting in greater hyperpolarization, delayed return to the resting potential of the mitral cells of the olfactory bulb and reduced excitability [57]. More specifically, there is a lower frequency of action potentials for an increased interspike interval. On the contrary, the T allele determines a lower functioning of Kv1.3 followed by a reduced outward potassium current, less negative membrane potential and greater nervous excitability.

Several studies on nutrition have shown that both the central and peripheral nervous systems are involved in the multiple and complex physiological mechanisms at the base of food intake [58,59]. In particular, a relationship between olfactory physiology/function and eating behavior has been reported [30]. On the one hand, metabolic imbalances can condition the olfactory function, and on the other, the olfactory function can affect the energy balance and body weight [30]. Given the relationship between *Kv1.3* polymorphism and smell and that between smell and body mass index [33], the second objective was to verify for a significant effect of the *rs2821557* polymorphism (*T*/*C*) of the human *Kv1.3* gene on the BMIs of individuals. The results we obtained show that subjects who were CC homozygous achieved BMIs that were significantly higher than those who were TT homozygous or TC heterozygous. In agreement, we found a different genotype distribution and different allele frequencies between subjects classified as normal weight or overweight: a lower BMI was associated with the genotype TT and allele T, and an overweight condition with the CC genotype and C allele. Previous data showed that *Kv1.3* mice exhibit increased insulin sensitivity, reduced plasma glucose concentrations [27,28,53] and resistance to diet-induced obesity [55], weighing significantly less than controls, despite the same amount of food ingested [56]. Xu and colleagues [56] suggested that Kv1.3 channels may participate in the pathway that regulates body weight, and that the inhibition of these channels increases basal metabolism and therefore weight loss. As a whole, our results show that subjects who carry at least one T allele not only have a better olfactory function, as demonstrated by the higher scores obtained, but also show a lower BMI. On the contrary, CC homozygous individuals show both a higher BMI and a reduced olfactory function. Considering also the negative correlations obtained between BMI and olfactory scores, both when individuals were considered all together and when they were divided according to sex, these data confirm that the olfactory perception of food can influence its selection. Most people who experience olfactory deficits report that food is both less tasty and less pleasant. They modify their eating behavior and, to compensate for the reduced pleasure in eating linked to the release of aromas in the oropharyngeal cavity, they increase food intake and thus experience weight gain [60,61,62,63]. A lower ability to discriminate and identify odors, as well as a lower perception of odors (olfactory threshold), is associated with the following nutritional risks: lower interest in food-related activities, such as cooking, or having a varied diet; less preference for foods with a bitter or acidic taste (which generally represent low-calorie foods); a higher intake of sweets (more calories); and an increased consumption of high-calorie foods [5,6,61,64,65].

One of the smell-related topics that is still a matter of debate concerns the differences related to sex; some studies report that females perform better, while others find no differences [35,37,66,67]. Furthermore, in recent years, research has been directed more to understand the causes of this difference than to finally shed light on its real existence [37]. Therefore, we evaluated the presence of differences in olfactory function linked to sex and studied for an involvement in these of the *rs2821557* (*T*/*C*) polymorphism of the human gene-encoding *Kv1.3* potassium channels. The results we obtained show that, for both sexes, individuals carrying at least one T allele achieved higher olfactory scores and showed lower BMIs than CC homozygotes. However, while no difference was observed between TT or CC homozygotes, in the case of TC heterozygotes we found that females achieved higher TDI, T and D olfactory scores than males and had lower BMIs. These results show that a T allele seems to protect females from olfactory dysfunction and weight gain, while males need to carry two T alleles to have olfactory and BMI performances comparable to those of females. Furthermore, when female and male individuals were also divided on the basis of their TDI olfactory status, the results show that the differences between TC females and TC males were associated only with the hyposmic state. In fact, no difference was found between subjects classified as normosmic, thus suggesting that a normosmia condition appears to balance the genetic effects linked to sex.

## 5. Conclusions

In conclusion, our results show that olfactory function and body weight are affected by the *rs2821557* (*T*/*C*) polymorphism of the human gene-encoding *Kv1.3* potassium channels. In fact, the olfactory system and its individual variability play important roles in the regulation of body weight, not only by acting on the quality and quantity of food that is ingested, but also on the energy and basal metabolism, through the activity of the voltage-gated potassium Kv1.3 channels. Given the differences between heterozygous males and females, we can hypothesize an involvement of this polymorphism of the human gene Kv1.3 in the physiological variability of olfactory function linked to sex. The results also highlight that the normosmia condition appears to balance the BMI differences shown between heterozygous males and females. Therefore, further studies on the individual variability of the olfactory function and its complex relationships with body weight, also in relation to sex, will be necessary to better understand which other mechanisms and factors (physiological, genetic and environmental) are involved, and how these are interconnected.

## Figures and Tables

**Table 1:** Genotype distribution and allele frequencies of the *rs2821557* polymorphism of the *Kv1.3* gene (*T/C*) in the subjects classified as normosmic or hyposmic on the basis of the TDI, T, D and I olfactory scores obtained.

		Normosmic *n* (%)	Hyposmic *n* (%)	*p*-Value
	Genotype			
TDI	TT TC CC	27 (57.55) 20 (42.55) 0 (0)	18 (32.73) 20 (36.56) 17 (30.91)	<0.0001
	Allele			
TDI	T C	74 (78.72) 20 (21.28)	56 (50.91) 54 (49.09)	0.0001
	Genotype			
T	TT TC CC	23 (47.92) 25 (52.08) 0 (0)	22 (40.74) 15 (27.78) 17 (31.48)	0.0088
	Allele			
T	T C	71 (73.96) 25 (26.04)	59 (54.63) 49 (45.37)	0.0055
	Genotype			
D	TT TC CC	42 (52.50) 36 (45.00) 2 (2.50)	3 (13.64) 4 (18.18) 15 (68.18)	1.19 × 10^−7^
	Allele			
D	T C	120 (75.00) 40 (25.00)	10 (22.73) 34 (77.27)	1.19 × 10^−7^
	Genotype			
I	TT TC CC	43 (49.43) 34 (39.08) 10 (11.49)	2 (13.33) 6 (40.00) 7 (46.67)	0.0010
	Allele			
I	T C	120 (68.97) 54 (31.03)	10 (33.33) 20 (66.67)	0.0004

*p*-value derived from Fisher’s exact test. Genotype: TT, *n* = 45; TC, *n* = 40; CC, *n* = 17.

**Table 2:** Genotype distribution and allele frequencies of the *rs2821557* polymorphism of the *Kv1.3* gene (*T/C*) in the subjects classified as normal weight or overweight on the basis of their BMI.

BMI	Normal Weight *n* (%)	Overweight *n* (%)	*p*-Value
Genotype			8 × 10^−5^
TT TC CC	37 (51.39) 31 (43.06) 4 (5.56)	8 (26.67) 9 (30.00) 13 (43.33)	
Allele			0.00015
T C	105 (72.92) 39 (27.08)	25 (41.67) 35 (58.33)	

*p*-value derived from Fisher’s method. Genotype: TT, *n* = 45; TC, *n* = 40; CC, *n* = 17.

## Data Availability

The data presented in this study are available on request from the corresponding author. The data are not publicly available due to restrictions (e.g., privacy or ethical).
